# Plutonic Rocks as Protection Layers to Concrete Exposed to Ultra-High Temperature

**DOI:** 10.3390/ma15103490

**Published:** 2022-05-12

**Authors:** Fernando França de Mendonça Filho, Cláudia Romero Rodriguez, Erik Schlangen, Oğuzhan Çopuroğlu

**Affiliations:** Materials & Environment, Faculty of Civil Engineering and Geosciences, Delft University of Technology, Stevinweg 1, 2628 CN Delft, The Netherlands; c.romerorodriguez@tudelft.nl (C.R.R.); erik.schlangen@tudelft.nl (E.S.); o.copuroglu@tudelft.nl (O.Ç.)

**Keywords:** dunite, microgabbro, thermal decomposition, stone-concrete composite

## Abstract

Concrete structures perform poorly when withstanding thermal shock events, usually requiring repair or replacement after one single instance. In certain industries (such as petrol, metallurgic and ceramics), these events are not only likely but frequent, which represents a considerable financial burden. One option to solve this issue would be to decrease the heating rate imposed onto the concrete material through the use of a protective surface layer. In this work, the suitability of dunite and microgabbro as protective materials is explored through X-ray diffraction, thermal dilation, optical microscopy, X-ray microtomography, thermo-gravimetric analysis and a compressive test. Further, the thermal dilation was used as an input to simulate a composite concrete-rock wall and the respective stresses caused by a thermal shock event. The dehydration of chrysotile in dunite and the decomposition of analcime, chamosite and pumpellyite in microgabbro were both favourable for the performance of the stones in the desired application. The thermal stability and deformation were found in the range of what can be applied directly on concrete; however, it was clear that pre-heating treatment results in a far more durable system in a cyclic thermal load situation.

## 1. Introduction

Concrete structures are weak against thermal shock events [1,2,3], needing to be replaced after a single instance [4,5,6]. Such events can be characterised by contact with substances at a thermal gradient as low as $\Delta T\geq 300{\;}^{{}^{\circ}}$C [7]. However, the environments being considered in the present research assume the spill of a product (e.g., molten slag, pig iron, unrefined oil, liquid aluminium) into an ambient temperature concrete structure. In this scenario, the thermal shock usually ranges from the temperature of concrete (from 5 ${}^{{}^{\circ}}$C to 40 ${}^{{}^{\circ}}$C) to the temperature of the product (from 800 ${}^{{}^{\circ}}$C to 1500 ${}^{{}^{\circ}}$C).

While not a major point of concern in the design of residential buildings, facilities for specific industries suffer from frequent thermal shock events and the accompanying need for repairs. Further, the constant replacement of a structure is time consuming and not environmentally sustainable. Yet, concrete is still the most used option for such structures given its low cost, relatively low specific weight and its ability to be cast into most desired geometries.

The response of solid materials to thermal shock can be theoretically described by Equation (1) [8] in which $\Delta T_{\mathrm{crit}}$ is the critical temperature gradient at which the material is expected to fail, *C* is a constant, $K_{\mathrm{IC}}$ is the toughness index of the material, *E* is the Young’s modulus, α is the coefficient of thermal expansion, *V* is the volume and $S_{a}$ is the surface area. Ordinary concrete presents a low toughness index and usually a high volume-to-surface ratio, hence its poor performance against thermal shock [8,9,10].

(1) $$\Delta T_{\mathrm{crit}}=\frac{C\cdot K_{\mathrm{IC}}}{E\cdot \alpha }\cdot {\left(\sqrt{\pi \cdot \frac{V}{S_{a}}}\right)}^{-1}$$

While the second term of the equation is governed by project specific requirements, the first term is largely dependent on the material design. Therefore, a high-performance mix can be selected to delay cracking at high temperatures. Yet, high-performance concrete behaves notoriously worse than ordinary concrete during thermal shock events [6,11,12]. This is because Equation (1) only accounts for the thermo-mechanical damage in solids, while concrete is a porous material.

Instead, the thermo-hydral damage mechanism can cause explosive detachment when the water vapour inside the pores of the material cannot readily escape [13,14,15]. This is governed by the availability of the connected pores in the material [16] and the heating rate at the surface [7]. Because of such a mechanism, porosity becomes essential for the durability of concrete. However, porosity is associated with a number of non-high-temperature-related concrete deterioration mechanisms [17,18,19,20].

A different approach would be to decrease the heating rate instead of changing the material properties. It was found that at fast heating rates, such as the ones in fire events, spalling occurs due to temperature-gradient-induced stresses, resulting in violent events [21]. However, at slower heating rates, the much smoother temperature gradient avoids a moisture clog, which would be the dominant form of spalling in these cases [22]. Some studies found this as the main difference between a slow loss of strength and explosive failure for given concrete mixes [16,21,22].

The problem lies in how to achieve the necessary decrease in the heating rate. Factories and industrial facilities rarely have the possibility of changing the operation temperature of their products; thus, a different solution must be sought. A protective surface layer better suited to withstand high-temperature spills could be added to the concrete structure. Such an overlay element would heat up and dissipate energy before the underlaying concrete can be affected and later slowly transfer the warmth to the substrate structure, greatly decreasing the danger of explosive spalling.

However, a number of conditions must be met before choosing a material for such an application. Because of the size of structures usually encountered in such facilities, the material would have to be available in large quantities and within reasonable geographical proximity. Furthermore, it would be desirable for it to possess a similar thermal dilation to concrete at low temperatures while displaying lower strains at high temperatures. It should also be chemically stable at high temperatures to allow for good durability when exposed to frequent events and possess high toughness to resist eventual impacts. Finally, a compressive strength similar or superior to the used concrete would be desirable.

This paper studies the viability of dunite and microgabbro as protective surface layers in concrete structures in order to protect them against high-temperature thermal shock events. The main research question pertains to the engineering properties of such rocks in their natural state and after being exposed to high temperatures and whether these would eventually be compatible with conventional concrete mixes.

These two rocks were pre-selected based on their geological properties and availability, as a starting point. Later, in order to investigate the mechanical and chemical properties, samples were prepared for thermogravimetric analysis, X-ray diffraction, thermal dilation, optical microscopy and X-ray microtomography. By looking at the chemical shift on the rocks after their heating and their respective microstructural response, conclusions were drawn regarding their adequacy for the task. Further, the thermal dilation was used as an input to simulate a composite concrete-rock wall and study the respective stresses caused by a thermal shock event.

## 2. Materials and Methods

### 2.1. Material Studied

**Figure 1:**
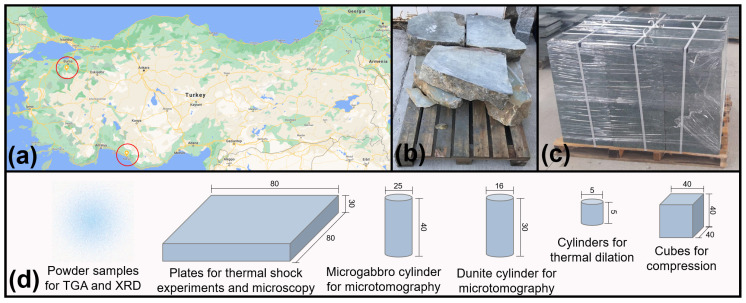
(**a**) Geographic location where the rocks were mined; (**b**) pallet of dunite; (**c**) pallet of microgabbro; (**d**) sub-sampling shapes and dimensions for experiments. All units in millimetres.

Dunite was mined from the Orhaneli ophiolite, situated in the western part of the Izmir–Ankara–Erzincan Suture Zone and south of Bursa (Turkey), an area that exposes cumulates belonging to the mantle–crust transition zone [23]. Microgabbro was mined from the Alanya Massif (Turkey), a microcontinent area that possesses ductile-deformed meta-basaltic rocks [24]. Both were cut for transportation purposes. The rocks were later sub-sampled for the experiments. Figure 1 shows an overview of the rocks’ location, appearance and sub-sampling for experiments. Prior to the experiments, half the samples were heated to a temperature of 900 ${}^{{}^{\circ}}$C for one hour. This allows for comparison of the behaviour of the non-heated rocks against the pre-heated stones.

### 2.2. X-ray Diffraction

In order to determine the mineralogical composition of the rocks, samples (in natural form and pre-heated state) were ground to fine powder and kept dry until testing through X-ray diffraction. A Bruker D8 Advance diffractometer Bragg–Brentano geometry and Lynxeye position sensitive detector was used with $CuK\alpha_{1,2}\left(\lambda =1.5408\right)$ radiation for all XRD measurements. The scatter screen height was set at 5 mm height, divergence slit V12. The used voltage was 45 kV with a current of 40 mA. The spin was of the sample. All scans were performed with a 2θ angle between 10${}^{{}^{\circ}}$ and 130${}^{{}^{\circ}}$, with a step size of 0.03${}^{{}^{\circ}}$. The counting time per step was two seconds. A combination of two software packages, X’Pert HighScore Plus and Jade6, was used for the spectral analysis. The standard software databases sufficed for peak identification. This method has been previously reported in Mendonca Filho et al. (2022) [25].

### 2.3. Thermal Gravimetric Analysis

Powder samples, prepared in the same fashion as described in Section 2.2, were used to perform thermogravimetric analysis (TGA) with the goal of understanding the chemical transformations in the rocks when heated. For the analyses, a NIETZSCH STA 449 F3 Jupiter was used. Approximately 40 mg of material was used for each analysis. The mass change of samples was continuously monitored while the temperature was raised at a 10 ${}^{{}^{\circ}}$C/m rate from 40 ${}^{{}^{\circ}}$C to 1000 ${}^{{}^{\circ}}$C. In order to perform the measurements, a 1 mm${}^{3}$ alumina crucible containing each sample was placed under a dry argon atmosphere with a purge rate of 50 mL/min, as advised in Scrivner et al. (2017) [26].

### 2.4. Optical Microscopy

To complement the results of X-ray diffraction while simulating a thermal shock event, optical microscopy was performed. For this test, plates of 80 mm of length and width with 30 mm of depth were cut. Each plate then was exposed to 40 g of molten slag heated at 1530 ${}^{{}^{\circ}}$C to simulate a thermal shock event. After the exposure, the samples were allowed to cool for 12 h, then the slag was removed, and the specimens were cut in half. From the halved vertical face, petrographic thin sections were produced. For this purpose, a controlled grinding process was employed with a semi-automatic thin sectioning machine, with three diamonds rollers of 64, 46 and 16 μm. Measurements with an electronic drop indicator yielded a thickness of about 22 μm cement paste and about 8 μm mounting glue. A more in-depth description of the sample preparation can be found in Poole and Sims (2016) [27].

The analysis was performed through a Leica DM2500P optical microscope equipped with linear and circular polarisation filters. Lenses of magnification and numerical aperture equivalent to 2.5/0.07, 10/0.22 and 20/0.4, respectively, were available. Plain polarised light (PPL) and cross polarised light (XPL) were used. The camera used to acquire the micrographs was a Leica DFC310FX digital camera, with a 1392 × 1040 resolution. Proprietary software was used for the image acquisition.

### 2.5. Microtomography

For the quantification of different components of the rocks, cylindrical samples of non-heated dunite and microgabbro were scanned by a Phoenix Nanotom X-ray CT scanner. These samples were then heated to a temperature of 900 ${}^{{}^{\circ}}$C at a rate of 10 ${}^{{}^{\circ}}$C/min and re-scanned using the same equipment. The scanner is composed of a transmission type X-ray tube, a sample stage and a 3072 × 2400 flat panel detector with a pixel size of 100 μm. The transmission target uses a tungsten filament and possesses a maximum accelerating voltage of 180 kV. However, the acquisitions were performed at 120 kV. A dunite sample of 16 mm diameter was used, resulting in a spatial resolution of 8 μm/pixel. The microgabbro cylinder had a 25 mm diameter, resulting in a spatial resolution of 13.33 μm/pixel. Different sample sizes were used because dunite presented fine cracks that could be better analysed using a higher spatial resolution. Meanwhile, microgabbro has relatively larger grains, which can be better studied with a larger field of view. Reconstruction was done through datos veloCt, the software provided by the equipment manufacturer. This procedure has been previously described by Rodriguez et al. (2020) [28].

Bruker’s DataViewer software was used for registration. The scanners of the natural stones could be registered to their pre-heated counterpart through true 3D registration using rigid transform, so expansion could also be detected. The post-processing of the images was performed using the freeware Fiji image analysis [29].

For dunite scans, a Gaussian blur filter (with 2 pixel radius) was used, followed by a non-local means denoising filter [30] in order to reduce noise. For phase segmentation, a classifier was trained using 3D trainable WEKA segmentation [31]. Because the segmentation of fine, crack-like phases is challenging, the authors opted to use a randomisable filtered classifier as basis for the WEKA training. For microgabbro, a median filter (with 2 pixel radius) was used, followed by the mentioned non-local means denoising filter. The segmentation of microgabbro samples was performed based on thresholding. The plugin 3D objects counter [32] was used to acquire the size distribution and diverse geometric properties of the phases after segmentation.

### 2.6. Thermal Dilation

Cylindrical cores were extracted from non-heated dunite, pre-heated dunite, non-heated microgabbro and pre-heated microgabbro. The dimensions of these were 5 mm in diameter and varying heights between 3 mm and 7 mm. A Perkin Elmer TMA 4000 thermomechanical analyser was used to determine the coefficient of thermal expansion (CTE) of the samples. The CTE was determined by heating each sample from ambient temperature (24 ${}^{{}^{\circ}}$C) to 800 ${}^{{}^{\circ}}$C at a heating rate of 2.8 ${}^{{}^{\circ}}$C/min and continuously monitoring the strain of the samples.

### 2.7. Compressive Strength

Three cubes with 40 mm sides were tested for each rock in order to determine compressive strength according to NEN-EN 1926:2007 [33]. Samples were tested in natural state and after the pre-heating treatment. The samples were tested at a fixed loading rate of 13.5 kN/s using a servo-hydraulic mechanical press with maximum load capacity of 5000 kN and high stability from Matest. Table 1 shows a review of shapes and dimensions of samples for each test.

### 2.8. Stress Comparison

A finite element software (COMSOL^®^ 5.3) was used to simulate the behaviour of a composite wall with a concrete substrate and a stone overlay exposed to hot spills. This analysis is performed to gain insight into the suitability of the pre-selected material as concrete overlay. The simulated concrete wall had 3 m in height, 10 m in length and 50 cm in depth. The stone element was created with equal height and length, but only 10 cm deep. A spill in one quarter of the wall was designed to create a sudden thermal shock from ambient temperature up to 1000 ${}^{{}^{\circ}}$C followed by the cooling of material based on previous research from the authors [34]. A 2D triangular mesh with maximum element size of 3 cm was used for the FEM simulation. The wall was considered fixed in the bottom and in the back, while the top and the face with the protective stone were free. The fixed faces were also considered to have thermal insulation. The concrete material was considered porous, while the rocks were set as solid materials. The temperature estimation was later used to couple the heat transfer and solid mechanics modules of the software for the calculation of stresses derived from the thermal expansion. While average concrete properties were used for such computations, the results of the stones’ analyses were fed as input.

## 3. Results

### 3.1. X-ray Diffraction

**Figure 2:**
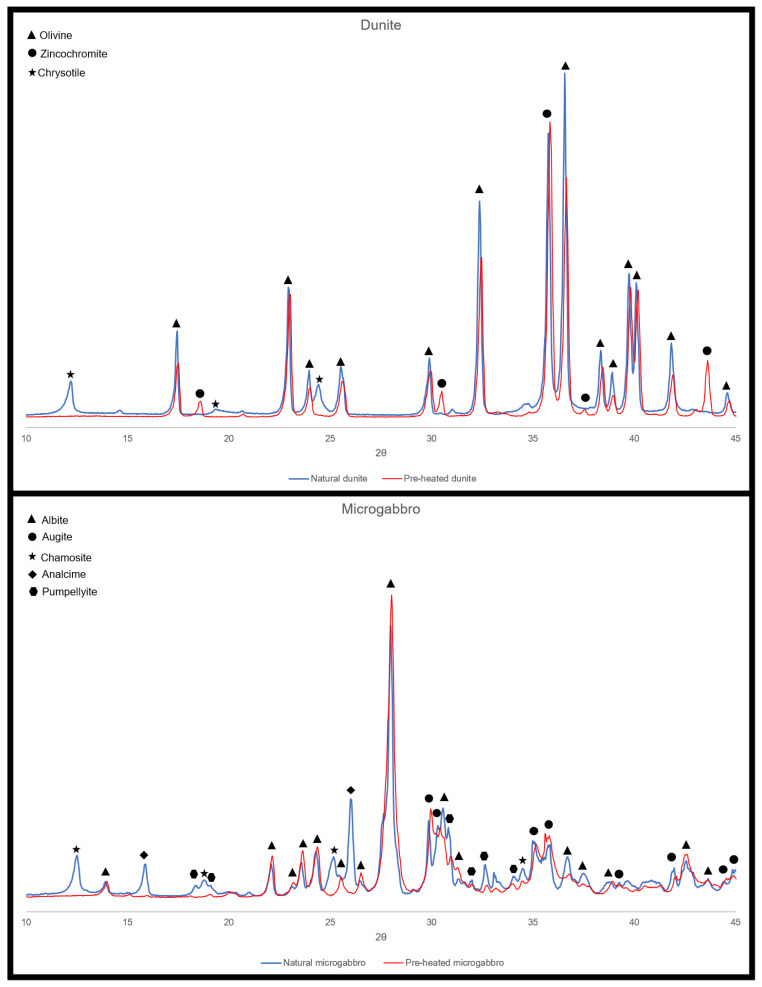
XRD results for dunite (**top**) and microgabbro (**bottom**) samples. The natural samples (in blue) are overlapped with their pre-heated counter parts (in red).

The results of the X-ray diffraction for the dunite and microgabbro can be found in Figure 2. As expected, the dunite is mostly composed of olivine, predominantly forsterite ($Mg_{2}SiO_{4}$). The best fit was found with a sample of the molecular percentage of fosterite to fayelite ($Fe_{2}SiO_{4}$) equivalent to $Fo_{95}Fa_{05}$. Zincochromite ($ZnCr_{2}O_{4}$) was the second most abundant mineral detected, although the peaks were less visible on the non-heated dunite sample. Trace amounts of chrysolite ($Mg_{3}\left(Si_{2}O_{5}\right){\left(OH\right)}_{4}$) were also identified, which is common due the serpentinisation of olivine [35,36]. At 900 ${}^{{}^{\circ}}$C, it is expected that the chrysolite will go through dehydration following Equation (2) [37], which is confirmed by the absence of peaks on the heated dunite sample.

(2) 2Mg3Si2O5(OH)4(serpentine)→3Mg2SiO4(forsterite)+SiO2(silica)+4H2O(fluid)

Microgabbro was found to be composed mostly of albite ($NaAlSi_{3}O_{8}$), with considerable amounts of chamosite (${\left(Fe,Al,Mg\right)}_{6}{\left(Si,Al\right)}_{4}O_{10}{\left(OH\right)}_{8}$) and pumpellyite ($Ca_{2}FeAl_{2}\left(SiO_{4}\right)$$\left(Si_{2}O_{7}\right){\left(OH\right)}_{2}\cdot H_{2}O$) and small amounts of aluminian augite ($Ca\left(Mg,Fe,Al\right){\left(Si,Al\right)}_{2}O_{6}$) and analcime ($Na\left(Si_{2}Al\right)O_{6}\cdot H_{2}O$). On the pre-heated sample, the peaks for chamosite, pumpellyite and analcime were absent. For analcime, this is explained due to the mineral dehydration at relatively low temperatures and pressure [38]. For pumpellyite, the dehydration occurs at even lower temperatures ($T<300{\;}^{{}^{\circ}}$C) [39], also in a single step. On the other hand, the behaviour of chamosite at higher temperatures is usually assumed to be a five-step reaction in which oxidation, dehydroxylation and decomposition generate $Mg\left(Si,Al_{2},Fe_{2}\right)O_{4}$ and amorphous $SiO_{2}$ [40].

### 3.2. Thermogravimetric Analysis

**Figure 3:**
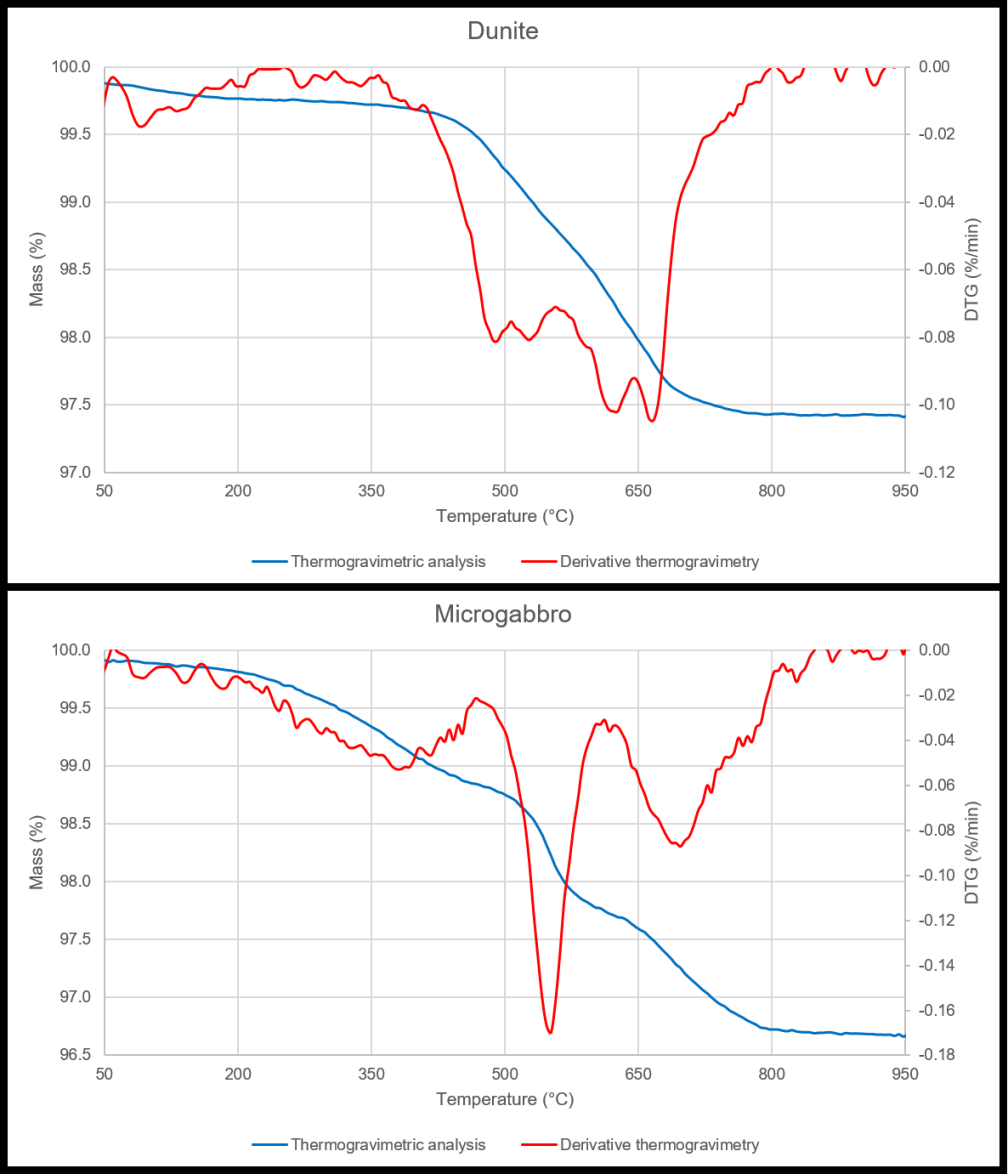
Thermogravimetric analysis results for dunite (**top**) and microgabbro (**bottom**) samples. The TGA results are seen in blue, while the DTG results are overlapped in red.

The results for the thermogravimetric analyses are presented in Figure 3. The small mass loss in dunite between 400 ${}^{{}^{\circ}}$C and 700 ${}^{{}^{\circ}}$C is attributed to the dehydration of the chrysolite [41]. On the other hand, three DTG peaks are present in the results for the microgabbro. The first peak, between 200 ${}^{{}^{\circ}}$C and 450 ${}^{{}^{\circ}}$C, corresponds to analcime [42], although with a very broad range of temperature, the maxima is characteristically located in the 350 ${}^{{}^{\circ}}$C–370 ${}^{{}^{\circ}}$C range [43]. The second peak (between 470 ${}^{{}^{\circ}}$C and 600 ${}^{{}^{\circ}}$C) and the third peak (between 615 ${}^{{}^{\circ}}$C and 800 ${}^{{}^{\circ}}$C) both belong to the thermal decomposition reactions of the chamosite [44]. Pumpellyite has a characteristic dehydration peak between 150 ${}^{{}^{\circ}}$C and 300 ${}^{{}^{\circ}}$C [39]; thus, it is overlapped with analcime.

### 3.3. Optical Microscopy

**Figure 4:**
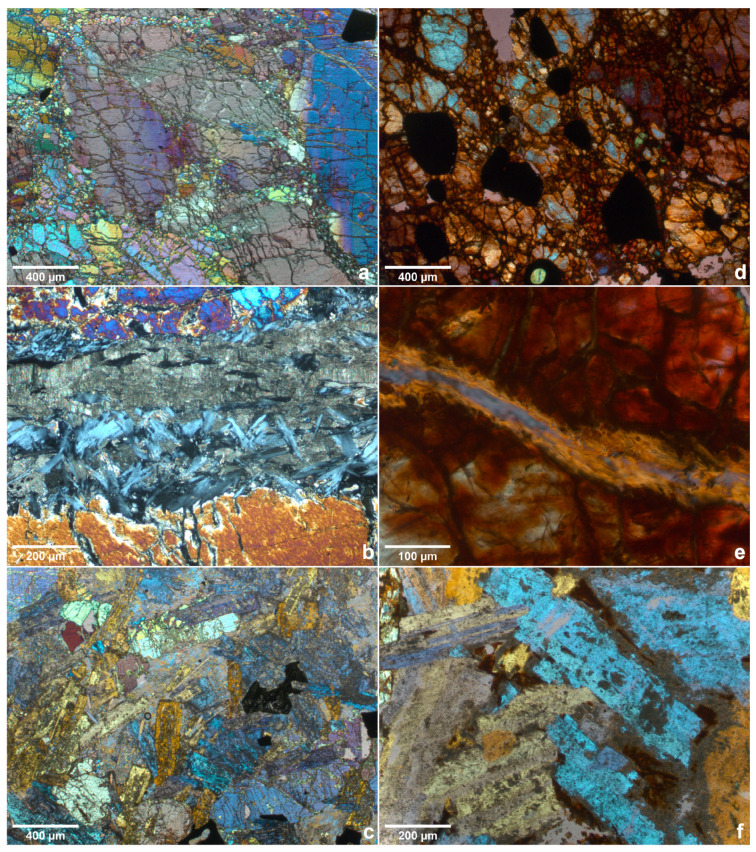
Thin sections of the samples: (**a**) natural dunite overview; (**b**) natural dunite, focus on chrysotile vein; (**c**) natural microgabbro overview; (**d**) pre-heated dunite overview; (**e**) pre-heated dunite, focus on chrysotile; (**f**) pre-heated gabbro overview.

Figure 4 shows some fields of view of the thin sections for all the samples. Based on the irregular extinction and banded structures in the large crystals, the olivine portion of dunite is assumed to be strained [45]. Furthermore, considering the interference colours present in Figure 4a, it can be assumed the olivine is magnesium rich [46]. The angular black features present in Figure 4a,d appear opaque in plain polarised light, as these are the zincochromite phase. Figure 4b highlights a strain of the chrysotile in the centre of the image with a dark grey fractured aspect. Embedded within the chrysotile, the brighter grey-bluish fibrous phase is antigorite, another mineral in the serpentine subgroup often identified by these features [47].

Figure 4d,e show a thin section made from the heated dunite sample, and the difference in interference colours is apparent. The reason for the red-brown to dark brown colour has been previously discussed in the works of Bron [48]. The colours are caused by the occurrence of hematite, which itself has two sources during the heating of dunite. The first being the development of dust-like separations of hematite by the olivine (which develops pleochroism); this is usually seen in the periphery of the grains. The second source was serpentine, oxidating $Fe^{2+}$ into $Fe^{3+}$. As expected, the chrysotile suffered dehydration after heating, losing its texture and leaving just vestiges of its presence. Figure 4e shows a previous site of chrysotile in the pre-heated sample.

The microgabbro displays a typical subhedral aspect with a phaneritic texture in Figure 4c, with pumpellyite apparent as brighter yellow and augite as deep purple. Both analcime [46] and chamosite [49] appear as opaque black, while albite can be seen in the centre, right of the image marked by a typical dark grey that is almost extinct [50]. The difference between the non-heated sample and the heated on transmission light microscopy is rather small, with Figure 4f showing only small details in the inter-granular space in red.

### 3.4. X-ray Microtomography

#### 3.4.1. Dunite

**Figure 5:**
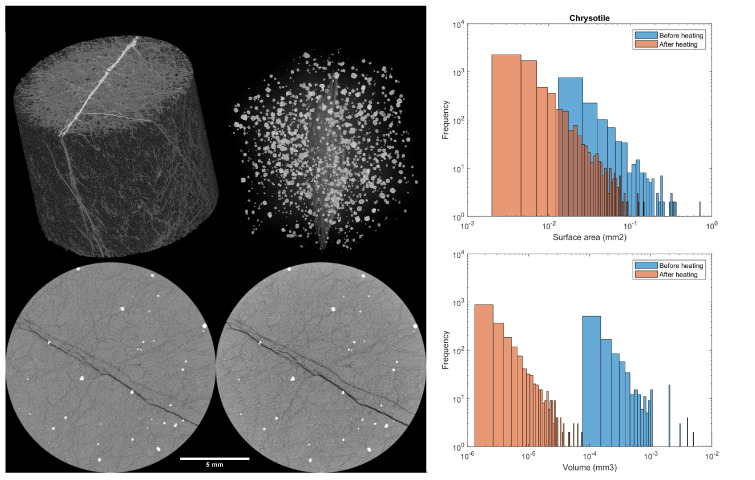
Results of dunite tomography. Top left: Representative volume studied. Top middle: Zincochromite and main serpentine vein. Bottom left: Slice of natural dunite. Bottom middle: Same slice after heating treatment. Top right: Changes in chrysotile surface area before and after heating treatment. Bottom right: Changes in chrysotile volume before and after heating treatment.

Through the registration process, it was clear that, after cooling, the heated samples return to their natural size, demonstrating good dimensional stability. The tomography of the dunite reveals three phases (in Figure 5), as expected by the XRD results. In the volumetric representation of Figure 5, serpentine is seen in white and light grey, while olivine is dark grey. In the 2D slices, zincochromite is shown in white, olivine in bright grey and chrysotile in dark grey and black. The phases can be clearly distinguished due to their density differences, as stated in Table 2.

According to Equation (2), the chrysotile dark grey and black phases should be eliminated after heating, which is confirmed by the XRD results. Yet, these phases remain visible in Figure 5. This is because the serpentine phase is transformed into forsterite and amorphous silica. While forsterite is indistinguishable from the original olivine phase in the tomography, the silica has a density of 2.196 g/cm${}^{3}$ [59] and the fluid is just 1 g/cm${}^{3}$. This means the dark grey and black phases in the scans are actually the silica and fluid generated by the conversion of the serpentine.

The serpentine conversion can be further inferred by the better contrast in the scan after heating (caused by the greater difference in the density of the phases) and by the 20% decrease in dark grey phases followed by the same proportional increase in the bright grey phases. This is a volume indication of how much forsterite and silica were generated from the change in the chrysotile. In accordance with the other tests, the zincochromite was not affected by the heating of the sample, remaining at approximately 0.7% in volume. The summary of the segmentation results can be found in Table 2.

Besides the total sample volume change, individual grains were investigated regarding their shift in surface area and volume. This is because the complex shape of the phases renders most geometrical measurements (i.e., diameter or bounding box) useless. Hence, only 3D information, such as the two measurements cited, could be reliably compared. As the zincochromite remained mostly unchanged after heating, the changes for the chrysotile are displayed in Figure 5.

#### 3.4.2. Microgabbro

**Figure 6:**
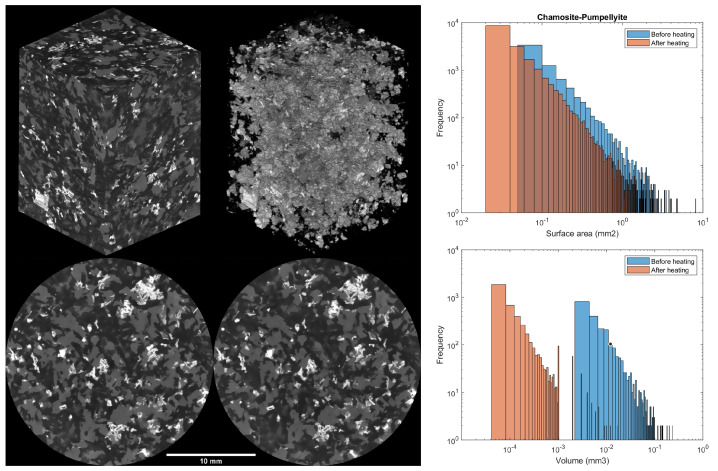
Results of microgabbro tomography. Top left: Representative volume studied. Top middle: Augite. Bottom left: Slice of natural microgabbro. Bottom middle: Same slice after heating. Top right: Changes in chamosite-pumpellyite surface area before and after heating treatment. Bottom right: Changes in chamosite-pumpellyite volume before and after heating treatment.

The tomography of the microgabbro only reveals three distinguishable phases (see Figure 6). Because the density of the analcime and albite are very similar, these two phases cannot be readily differentiated based on the digital volume; these were simply segmented as the darkest phase. The bright grey phase is a combination of pumpellyite and chamosite, also not possible to discriminate. Finally, the brighter phase in the slices is augite. The relative amounts and densities can be found in Table 2. The net transformation of the pumpellyite, chamosite and analcime decreased the volume of the bright grey phase by roughly 3%, which was increased in the dark grey phase. This can also be observed in the differences in the shape at the edges of the individual grains on the tomography slices.

The analcime was indistinguishable from the albite; thus, it could not be analysed. The augite was not affected by the heating treatment. However, the changes in the pumpellyite and chamosite display a far larger shift in volume than in surface area (see Figure 6). As expected, the dehydration and dehydroxilation happened from the grain surface inward, which caused the decrease in surface area for the three phases studied (including both dunite and microgabbro). Because chrysotile has a finer and acicular habit, the surface area was affected to a greater extent than the chamosite and pumpellyite for the same high-temperature exposure. For the volume, much of the decrease was caused by the separation of particles, which were considered a single entity before heating, evident in all three phases. Moreover, the difference between the shift in volume and surface area was a good indication of the shape change of the particles.

### 3.5. Compressive Strength, Thermal Dilation and Stresses Simulation

The results for the compressive strength are displayed in Table 3. The studied dunite presented a compressive strength comparable with high-performance concrete, which ensures the possibility for structural use. An increase of 77% in strength was observed after heating, pointing to a good durability for the selected application. The microgabbro compressive strength was found to be comparable with ultra-high-performance concrete; however, a decrease of 14% was registered after heating. As the strength after this decrease remained superior to 160 MPa, such a decrease was considered negligible.

**Figure 7:**
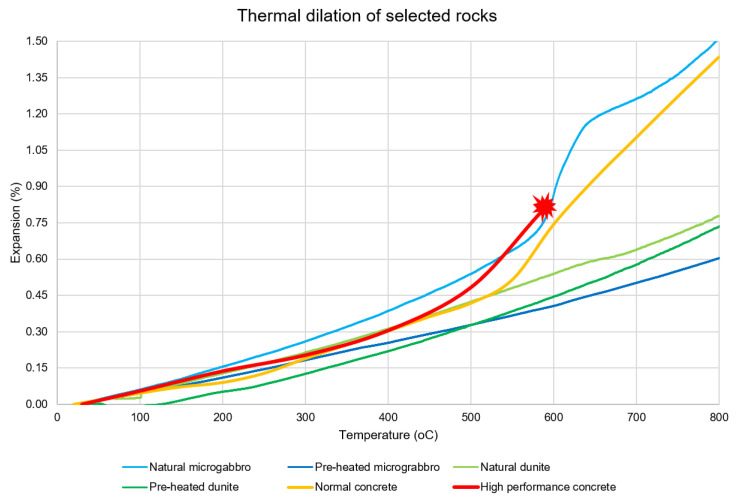
Thermal dilation of samples in natural and pre-heated state. Normal and high-strength concrete dilation results are added for comparison (the red line stops at the point the samples spalled).

Figure 7 shows the results for the thermal dilation of the samples. The available results for normal strength [60] and high-performance concrete [61] were added for comparison purposes. At temperatures below 100 ${}^{{}^{\circ}}$C, all samples possess thermal strain similar to concrete, with the exception of pre-heated dunite, which presents very low dilation. Between 100 ${}^{{}^{\circ}}$C and 500 ${}^{{}^{\circ}}$C, the natural microgabbro dilates more than an average concrete sample, while both pre-heated samples show a smaller dilation. Non-heated dunite shows a remarkably similar dilation. Above 500 ${}^{{}^{\circ}}$C, concrete usually has a sharp increase in dilation and damage accumulation. Natural microgabbro follows the dilation increase, matching the concrete well. The other stones remain linear, displaying good expansion at high temperatures.

Besides the increase in expansion, the difference between the expansions of non-heated and pre-heated dunite were almost linear, only diminishing at temperatures higher than 700 ${}^{{}^{\circ}}$C. In contrast, while non-heated microgabbro presents the non-linear expansion with a sudden increase between 550 ${}^{{}^{\circ}}$C and 650 ${}^{{}^{\circ}}$C, the pre-heated stone presents a linear expansion with a lower rate of expansion than dunite at temperatures higher than 500 ${}^{{}^{\circ}}$C.

**Figure 8:**
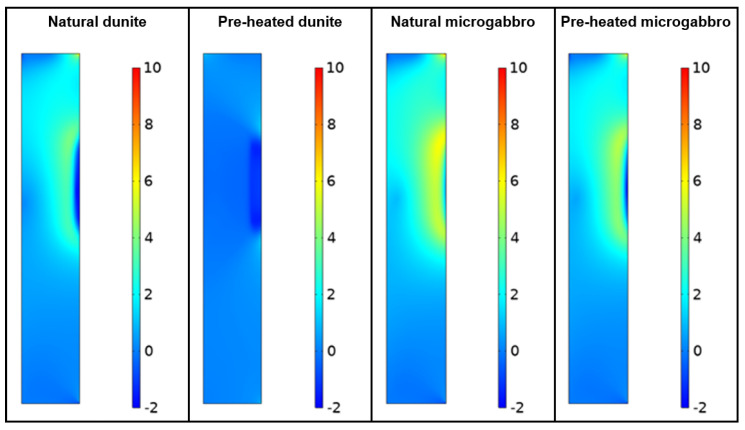
Principal stresses in simulated concrete walls at 50 min after hot spill exposure (scale in MPa).

Due to the stones capacity for energy absorption and the amount of molten material simulated, the temperature in the concrete wall layer can be kept below 100 ${}^{{}^{\circ}}$C, with the maximum temperature in the concrete occurring about 50 min after the exposure of the stone layers to the molten material. Figure 8 shows the stresses distribution on the cross-section of the simulated concrete walls at the 50 min time.

## 4. Discussion

In the dunite samples, the conversion of the chrysotile to forsterite and silica, as theorised in Equation (2) and indicated in the XRD and TGA results, seems to improve both the mechanical and thermal properties of the stone. From a mechanical point of view, such results were unexpected, as early studies showed the weakening of dunite by serpentine dehydration [62]. However, a more recent study [63] presents different conclusions in which the absence of pore fluid (through evaporation) and even strain-hardening during distributed deformation makes the influence of serpentine on the strength ambiguous. Although the mentioned study used antigorite as a reference, similar behaviour can be expected from chrysotile. Furthermore, small amounts of antigorite were detected as seen in the optical microscopy results. Another possible contributing factor could be the filling of spaces with hematite, as observed in the previous section.

Regarding the thermal dilation, both non-heated and pre-heated dunite show similar behaviour at a low temperature. After 100 ${}^{{}^{\circ}}$C, non-heated dunite expands an average of 10% more than pre-heated dunite. This value is proportional to the amount of chrysotile in the sample; in fact, the value changes at temperatures higher than 640 ${}^{{}^{\circ}}$C, when chrysotile is known to start losing structural water [64]. Although the non-heated dunite thermal expansion becomes more similar to its pre-heated twin after such temperatures, the latter still displays lower expansion due to its already reached mineral stability.

The small decrease in the microgabbro ultimate compressive strength was likely due to the small increase in porosity in its microstructure introduced by the conversion of pumpellyite, chamosite and analcime into magnesium oxide and $SiO_{2}$ (as corroborated by the difference in particle volume shown in Figure 6).

Although the observations through optical microscopy and microtomography both displayed limited changes in the microstructure, the change in the samples’ expansion caused by thermal loading was considerable. Unlike the dunite samples, the microgabbro samples presented a complex behaviour in which the difference between the natural and the pre-heated samples starts as early as 50 ${}^{{}^{\circ}}$C and increases at a second degree rate up to 570 ${}^{{}^{\circ}}$C.

In line with the results of the DTG and the findings of Caillére and Hénin [65], the steep increase in thermal expansion of the natural microgabbro between 570 ${}^{{}^{\circ}}$C and 640 ${}^{{}^{\circ}}$C was caused by the dehydroxylation of the chamosite. Because of this temperature interval, it is also clear that the chamosite is rich in Mg-hydroxy in its interlayer [65].

It seems clear that chamosite plays a dominant role in the thermal expansion behaviour of the studied microgabbro samples. As its full decomposition is not finished until temperatures are slightly above 800 ${}^{{}^{\circ}}$C [66], the performance observed in the pre-heated sample can only be expected after heating at 900 ${}^{{}^{\circ}}$C or above for a considerable amount of time.

As the stresses in the concrete (shown in Figure 8) were derived from the rocks’ expansion, smaller coefficients of thermal dilation are clearly advantageous for the protection layer. Because the concrete remains at relatively low temperatures (<200 ${}^{{}^{\circ}}$C), the pre-heated dunite material displays the least amount of principal stresses. In situations in which it is expected for the concrete to reach temperatures higher than 500 ${}^{{}^{\circ}}$C, a protection layer of stone could still secure a slower temperature increase (to avoid spalling). In this case, pre-heated microgabbro would likely perform better given its lower thermal expansion at such temperatures. In both cases, the pre-heated material presents clear advantages against its natural counterparts.

## 5. Conclusions

The present study proposed dunite and microgabbro as possible building materials to be used as a protective surface layer to concrete structures that are repeatedly exposed to high temperatures in industrial environments or otherwise. The main function of such surface layers would be to endure the thermal shock present in such environments and transfer the heat to the concrete at a much slower rate, reducing the risk of concrete spalling. To achieve this goal, the microstructure, mechanical properties and the thermal performance of these minerals have been studied.

The main changes in dunite were caused by the dehydration of chrysotile, which provides improvements in both mechanical and thermal properties. The structural changes in microgabbro were more complex, involving the decomposition of analcime, pumpellyite and chamosite, the latter only becoming complete after 800 ${}^{{}^{\circ}}$C. While the ultimate compressive strength slightly decreases, its thermal expansion improves considerably after heating.

The authors found that both rocks present befitting properties to their use as a protection layer in industrial applications. However, it is clear that pre-heating results in a far more durable system in a cyclic thermal load situation. Furthermore, the increase in strength in dunite was previously missing in the literature and requires further investigation.

## Figures and Tables

**Table 1:** Shape and dimensions of samples used for each test.

Analysis Method	Sample Shape	Sample Dimensions (mm)
X-ray diffraction	Powder	<0.01
Thermogravimetric analysis	Powder	<0.01
Optical microscopy	Rectangle mounted in glass	30 × 45 × 0.022
Compressive strength	Cube	40 × 40 × 40
Thermal dilation	Cylinder	5 × 5
Micro tomography (dunite)	Cylinder	16 × 30
Micro tomography (microgabbro)	Cylinder	25 × 40

**Table 2:** Relative amount of phases for dunite and microgabbro as computed from CT segmentation.

Phase	Density (g/cm${}^{3}$)	Volume before Heating (%)	Volume after Heating (%)
Zincochromite	5.434 [51]	0.66	0.69
Olivine	3.287 [52]	86.44	88.77
Chrysotile	2.56 [53]	11.53	9.33
Augite	3.4 [54]	07.76	07.37
Pumpellyite	3.18 [55]		
Chamosite	3.13 [56]	31.98	29.27
Albite	2.4 [57]		
Analcime	2.3 [58]	60.26	63.36

**Table 3:** Compressive strength results for dunite and microgabbro.

Sample	Compressive Strength (MPa)	Standard Deviation (MPa)
Dunite	84	8.58
Dunite (heated)	149	22.46
Microgabbro	193	4.31
Microgabbro (heated)	166	2.96

## Data Availability

Data sharing is not applicable to this article.

## Abbreviations

The following abbreviations are used in this manuscript:

XRD	X-ray diffraction spectroscopy
TGA	Thermal gravimetric analysis
DTG	Differential thermogravimetric analysis
PPL	Plain polarised light
XPL	Cross polarised light
CT	Computer tomography
WEKA	Waikato Environment for Knowledge Analysis
CTE	Coefficient of thermal expansion
FEM	Finite element model
